# Bilateral bartholin’s gland abscesses in a 4-year-old girl with vitamin a deficiency: a case report

**DOI:** 10.1186/s12879-024-09382-1

**Published:** 2024-05-11

**Authors:** Tomoko Kihara, Tsuneaki Kenzaka, Tomohiro Hasegawa, Koutaro Uemura, Toru Funakoshi

**Affiliations:** 1 Department of Obstetrics, Hyogo Prefectural Kobe Children’s Hospital, 1-6-7, Minatojimaminamimachi, Chuo-ku, Kobe, Hyogo 650-0047 Japan; 2 Department of Internal Medicine, Hyogo Prefectural Tamba Medical Center, Tamba, Japan; 3 Division of Community Medicine and Career Development, Kobe University Graduate School of Medicine, Kobe, Japan; 4 Department of General Medicine, Hyogo Prefectural Kobe Children’s Hospital, Kobe, Japan; 5 Department of Pediatric Surgery, Hyogo Prefectural Kobe Children’s Hospital, Kobe, Japan

**Keywords:** Bartholin’s gland abscesses, Infant, *Streptococcus constellatus*, *Prevotella bivia*, Case report

## Abstract

**Background:**

A Bartholin’s gland abscess is one of the most common infections in women of reproductive age. Although Bartholin’s gland abscesses have been reported in prepubertal children, they are rarer in prepubertal children than in adults. Herein, we report a case of bilateral Bartholin’s gland abscesses in a 4-year-old girl with vitamin A deficiency.

**Case presentation:**

A 4-year-old girl diagnosed with autism spectrum disorder was admitted to the hospital for close examination and treatment because of persistent fever and malaise. The child was a marked fussy eater and was diagnosed with corneal ulceration and night blindness secondary to vitamin A deficiency. Both of the patient’s labia were swollen, and a diagnosis of a bilateral Bartholin’s gland abscess was made using computed tomography. Incisional drainage was performed under general anesthesia. The patient’s postoperative course was uneventful, and she was discharged from the hospital on day 8 after the surgery. During hospitalization, attempts were made to correct the vitamin deficiency by adding nutritional supplements to the diet. Three months after the surgery, no recurrence of abscesses was noted.

**Conclusions:**

Decreased immunocompetence and mucosal barrier function due to vitamin A deficiency is thought to be the underlying cause of Bartholin’s gland abscesses. Although prepubertal Bartholin’s gland abscesses have been reported, they are rare. To the best of our knowledge, no reports of bilateral Bartholin’s gland abscesses potentially caused by vitamin A deficiency have been reported. When prepubertal girls present with Bartholin’s gland abscesses, the presence of immunodeficiency due to vitamin or trace element deficiency should also be considered.

## Background

A Bartholin’s gland abscess is one of the most common infections among sexually active women [1]. In women, the Bartholin’s glands are located on the inside of the labia; they secrete a fluid during sexual activity that moistens the vagina, prevents bacterial invasion, and acts as a lubricant during sexual intercourse [1]. Bartholin’s gland abscesses can be treated with antimicrobials and incisional drainage. However, the formation of an abscess can be quite painful, sometimes impeding walking and affecting quality of life. For instance, patients may find it difficult to sit in a chair owing to severe pain.

Obstruction of the orifice leads to a Bartholin’s gland cyst, and further bacterial infection results in a Bartholin’s gland abscess [1]. It is unlikely to occur before puberty when the Bartholin’s glands have not yet begun to secrete.

Although Bartholin’s gland abscesses in prepubertal girls have been reported [1–10], they are rarer in prepubertal girls than in adult women. Herein, we report a case of a bilateral Bartholin’s gland abscesses in a 4-year-old girl with vitamin A deficiency.

## Case presentation

A 4-year-old girl with autism spectrum disorder was hospitalized for close examination and treatment following the development of a fever and gradual poor oral intake. The patient’s height was 91.5 cm, and her weight was 14.5 kg. When spoken to, she did not make eye contact or respond. Her right eyeball was cloudy because of a corneal ulceration.

She was a fussy eater, eating only French fries from a particular fast-food company. The patient defecated in a diaper. At 4 years of age, 3 months before the current admission, she was diagnosed with a right corneal ulcer, left upper eyelid chalazion, and night blindness due to vitamin A deficiency, for which antimicrobial (cephem type 3) and hyaluronic acid eye drops were administered.

The patient’s body temperature at the time of transfer was 38.7 °C, blood pressure was 100/77 mmHg, heart rate was 100 beats/min, and oxygen saturation was 98% (room air). Upon admission, blood test results revealed a leukocyte count of 21,600 cells/µL, hemoglobin concentration of 10.1 g/dL, C-reactive protein concentration of 6.57 mg/dL, and elevated inflammatory reactions, whereas urinalysis revealed cloudy urine and the presence of bacteria. Owing to the patient’s history of refractory cystitis, she was prescribed 700 mg piperacillin (50 mg/kg) for a suspected urinary tract infection.

**Fig. 1:**
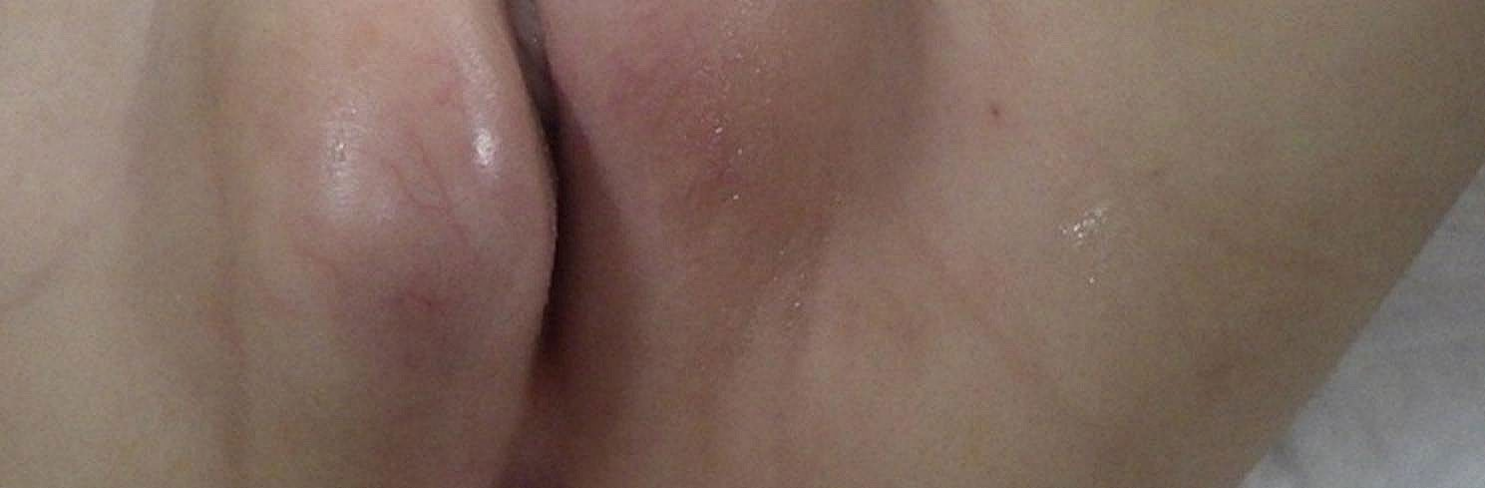
Before drainage swollen labia majora on both sides

**Fig. 2:**
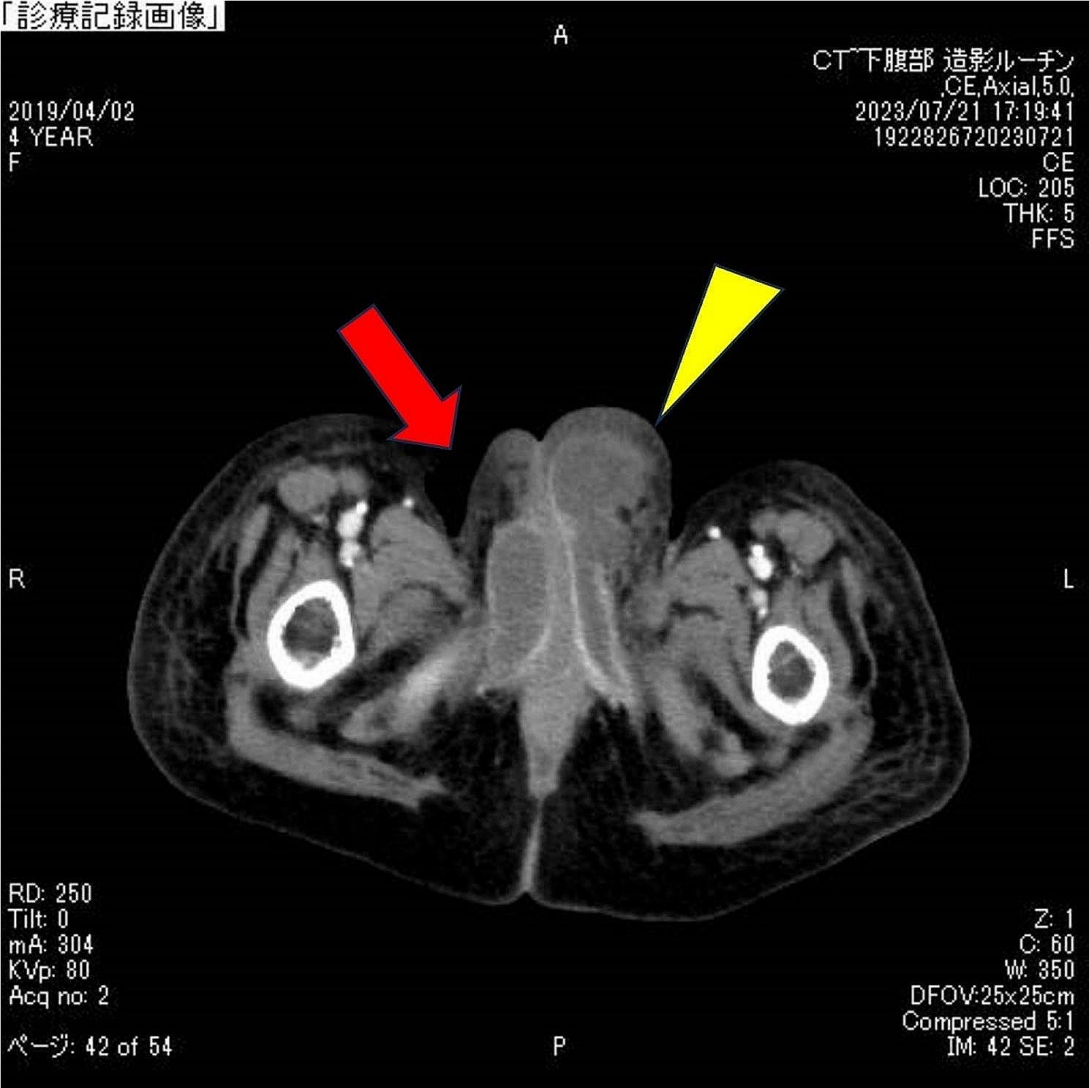
Contrast-enhanced computed tomography enhanced and swollen labia on both sides indicating bilateral bartholin’s gland abscesses (red arrow and yellow triangle)

On day 9 of admission, the patient exhibited bilateral swelling of the labia majora, predominantly on the left side (Fig. 1). Ultrasonography revealed a subcutaneous abscess, superficial echocardiography revealed fluid retention in the left labia majora over the inguinal area, and contrast-enhanced computed tomography revealed fluid accumulation with a contrast effect in the labia majora. Therefore, a diagnosis of bilateral Bartholin’s gland abscesses was made (Fig. 2), and the patient was referred to the pediatric surgery and obstetrics department for incision and drainage. Following maximum drainage of the abscess in the left labia majora, which was punctured with an 18-G needle, the contents were submitted for culture examination.

The blood test results were as follows: leukocytes, 36,300 cells/µL; hemoglobin, 8.9 g/dL; C-reactive protein, 2.57 mg/dL; *total protein, 6.4 g/dL; serum albumin, 3.1 g/dL;* ferritin, 112.2 (reference range, 3.6–114) ng/mL; iron, 13 (40–188) µg/dL; zinc, 40 (80–130) µg/dL; vitamin A, 6 (97–316) IU/dL; vitamin C, 2.9 (5.5–16.8) µg/ mL; and selenium, < 2.0 (107–171) µg/L.

The patient had an excessive inflammatory response, vitamin A and C deficiencies, and a trace element deficiency due to an unbalanced diet. Bacterial culturing of the abscess yielded anaerobic gram-positive cocci (streptococci), and intravenous treatment with 1,574 mg piperacillin/tazobactam (100 mg/kg) was initiated. Subsequently, *Streptococcus constellatus* and *Prevotella bivia* were detected in the abscess culture.

The swelling did not decrease after the abscess was punctured; thus, 1 week after the antimicrobials were administered, incisional draining of the abscesses was performed under general anesthesia, and the wounds were sutured completely to avoid dead space. The patient was intravenously treated with 4,350 mg sulbactam/ampicillin (300 mg/kg) until postoperative day 5 and discharged from the hospital on postoperative day 8. As she recovered without any complications, no further postoperative blood tests were performed.

An outpatient examination 1 week after discharge from the hospital revealed healing of the wound. Three months after the patient’s surgery, no recurrence of abscesses was noted. Although the patient’s picky eating seemed to have lessened after discharge from the hospital, she had already returned to eating only French fries 3 months after surgery and continued to receive treatment with nutritional supplements.

Initially, her parents demonstrated efforts to address her selective eating habits. However, over time, she exclusively consumed a specific brand of French fries, insisting on a particular location and plate for her meals. The child’s behavior had to be addressed before she succumbed to further malnutrition-related ailments and to provide support to families facing similar challenges within society. Hospitalization facilitated the parents’ comprehension of their child’s health impact and allowed for the monitoring of her nutritional status through the administration of supplements and the assessment of trace elements and vitamins as required.

## Discussion

Bartholin’s gland abscesses are known to occur in sexually mature female individuals. That is why, for a prepubertal child, Bartholin’s gland abscess is normally excluded from the differential diagnosis. In Table 1, we summarize previous case reports of Bartholin’s gland abscesses in prepubertal children.

**Table 1:** List of cases of prepubertal bartholin’s gland abscess

No.	Reference No.	Author	Year	Age	Treatment	Culture results	Complications (mother’s infection in the neonate)
1	3	El Kady et al.	2007	1 month	I & D + A	Negative	None
2	8	Chavarría et al.	1989	3 days	I & D + A	*Escherichia coli*	Bacterial vaginitis in the mother: trichomoniasis
3	11	Schauffler et al.	1939	6.5 weeks	I & D	Unknown	None
4	12	Kubitz et al.	1986	5 weeks	I & D + A	*E. coli, Peptococcus*	Bacterial vaginitis in the mother: gonorrhea, chlamydia
5	13	Ernst et al.	1988	3 months	I & D + A	*E. coli, Klebsiella pneumoniae*	None
6	1	Singh et al.	2010	3 months	I & D + A	*E. coli*	None
7	7	Cevik et al.	2012	2 days	I & D + A	Not performed	Congenital anomalies of the kidney and urinary tract
8	2	Radhakrishna et al.	2017	7 years	I & D + A	Negative	None
9	9	Revathi et al.	2017	8 months	I & D + A	*Staphylococcus*	None
10	10	Popovic et al.	2018	11 years	I	Not performed	None
11		Present case	2023	4 years	I & D + A	*Streptococcus constellatus, Prevotella bivia*	Vitamin A deficiency, autism spectrum disorder

A: Antibiotics; I & D: Incision and Drainage

Bartholin’s gland cyst has been reported in a neonate with developmental abnormalities of the vaginal and urinary tract system [7]. However, vulvar masses in children are difficult to detect unless the patient complains about them. In this case, the child was on the autism spectrum and had difficulties communicating. She had an elevated inflammatory response that could not be explained by a urinary tract or upper respiratory infection, and a vulvar abnormality was first noticed during a diaper change.

The differential diagnosis included a lipoma, fibrosis, and infection of the canal of Nuck; however, imaging revealed a Bartholin’s gland abscess. Lipomas and fibromas typically present as solid masses and are rarely associated with infection. The canal of Nuck represents a cystic lesion extending into the inguinal region, which was ruled out based on imaging.

If local swelling and inflammation cannot be controlled with antimicrobial treatment already in place, incisional drainage is the treatment of choice for Bartholin’s gland abscesses. Considering that this disease can occur in girls who are not sexually mature, surgical treatment should be initiated at an appropriate time.

Our patient had a vitamin deficiency, particularly that of vitamin A, due to an unbalanced diet. Vitamin A deficiency has been associated with infectious diseases, particularly in developing countries, and reportedly contributes to conditions such as diarrhea, malaria, and severe cases of measles [11].

In Japan, as in other developed countries, few cases of vitamin deficiency are encountered. However, the nutritional situation should be considered for patients with certain behavioral tendencies, such as those with autism spectrum disorder.

Typical vitamin A deficiency disorders include corneal xerosis; night blindness; dryness, thickening, and keratinization of the skin; and infections due to decreased immunocompetence and dryness of the mucosal epithelium [12, 13].

Reports of infections acquired in the womb of a mother with a history of treatment for bacterial vaginitis have been documented [8, 13]. Furthermore, respiratory infections have been studied in infants with vitamin A deficiency [12]; however, we are not aware of other reports of bilateral Bartholin’s gland abscesses in the context of vitamin A deficiency. Vitamin A deficiency can result in immunodeficiency, rendering patients susceptible to infections [14]. This can result in infections that are less common in infants, such as that observed in this case. Although one does not immediately suspect a Bartholin’s gland cyst from a swollen vulva in a child, vitamin A deficiency due to an unbalanced diet may cause dry mucosal epithelium, facilitating bacterial infection of the vulva through excretion in the diaper. Other vitamin deficiencies have been linked to infectious diseases. For instance, vitamin C deficiency has been associated with a significant difference in mortality within 28 days among patients with sepsis in the intensive care unit, suggesting that vitamin deficiency plays a crucial role in infection control [15].

A Bartholin’s gland abscess is a disease that predominantly affects postpubertal female individuals but may also occur in infants and children under the conditions described above. In cases of unusual infectious diseases, the scope of the differential diagnosis should be expanded to consider deficiencies in trace elements and vitamins regardless of the patient’s age. For infants and other children who face challenges in communicating with caregivers, obtaining detailed information about their daily lives from parents is essential.

## Conclusions

Herein, we report a case of bilateral Bartholin’s gland abscesses in a child. In Japan, as in other developed countries, few cases of vitamin deficiency are encountered. Moreover, for prepubertal children, a Bartholin’s gland abscess is typically excluded from the differential diagnosis. However, for children with certain nutritional tendencies, such as those who are picky eaters, one should consider nutritional deficiencies when making a diagnosis. Vitamin A deficiency leads to immunodeficiency, and thus, increased susceptibility to infections is considered a contributing factor.

## Data Availability

All data generated or analyzed during this study are included in this published article.

## References

[1] Singh JK, Viruthagiri A, Sadasivan J (2010). Bartholin’s gland abscess–a rarity in infants and children. Curr Pediatr Res.

[2] Radhakrishna V, Goel R, Parashar G, Santhanakrishnan R (2017). Bartholin’s gland abscess in a prepubertal female: a case report. Ann Med Surg (Lond).

[3] El Kady S, Al Zahrani A, Jednak R, El Sherbiny M (2007). Bartholin’s gland abscess in a neonate: a case report. Can Urol Assoc J.

[4] Title of subordinate document. In: Title of main document. Publisher. Year of publication. https://www.mhlw.go.jp/file/05-Shingikai-10901000-Kenkoukyoku-Soumuka/0000042635.pdf. (Author name(s), Japanese.). Accessed 13 Mar 2024.

[5] Amimo JO, Michael H, Chepngeno J, Raev SA, Saif LJ, Vlasova AN (2022). Immune impairment associated with vitamin a deficiency: insights from clinical studies and animal model research. Nutrients.

[6] Abdelkader A, Wahba AA, El-Tonsy M, Zewail AA, Shams Eldin M (2022). Recurrent respiratory infections and vitamin A levels: a link? It is cross-sectional. Med (Baltim).

[7] Cevik M, Savas M, Guldur ME, Boleken ME (2012). Urinary retention as the presentation of Bartholin’s duct cyst in a neonate. J Pediatr Adolesc Gynecol.

[8] Chavarría JF, Faingezicht I (1989). Bartholin’s gland abscess in a neonate. Pediatr Infect Dis J.

[9] Revathi N, Pandey A, Patra V, Seth B (2017). Bilateral vulvar abscess in an infant–an unusual occurrence. New Indian J Pediatr.

[10] Popovic N, Zvizdic Z, Milisic E, Jonuzi A, Karamustafic A (2018). Large Bartholin’s gland cyst in a premenarchal girl: a rare clinical finding. Iran J Pediatr Surg.

[11] Mayo-Wilson E, Imdad A, Herzer K, Yakoob MY, Bhutta ZA (2011). Vitamin A supplements for preventing mortality, illness, and blindness in children aged under 5: systematic review and meta-analysis. BMJ.

[12] Schauffler GC, Kanzler R, Schauffler C (1939). Management of 256 cases of infection of the immature vagina. JAMA.

[13] Kubitz R, Hoffman K (1986). Bartholin’s gland abscess in an infant. A case report. J Reprod Med.

[14] Ernst EA, Weller P, Karch SB (1988). Bartholin’s gland abscess in infancy. Pediatr Infect Dis J.

[15] Lamontagne F, Masse M-H, Menard J, Sprague S, Pinto R, Heyland DK (2022). Intravenous vitamin C in adults with sepsis in the intensive care unit. N Engl J Med.

